# Patients’ understanding of cellulitis and their information needs: a mixed-methods study in primary and secondary care

**DOI:** 10.3399/bjgp19X701873

**Published:** 2019-03-12

**Authors:** Emma Teasdale, Anna Lalonde, Ingrid Muller, Joanne Chalmers, Peter Smart, Julie Hooper, Magdy El-Gohary, Kim S Thomas, Miriam Santer

**Affiliations:** Primary Care and Population Science, Faculty of Medicine, University of Southampton, Southampton.; Primary Care and Population Science, Faculty of Medicine, University of Southampton, Southampton.; Primary Care and Population Science, Faculty of Medicine, University of Southampton, Southampton.; Centre for Evidence Based Dermatology, University of Nottingham, Nottingham.; Primary Care and Population Science, Faculty of Medicine, University of Southampton, Southampton.; Centre for Evidence Based Dermatology, University of Nottingham, Nottingham.; Primary Care and Population Science, Faculty of Medicine, University of Southampton, Southampton.

**Keywords:** cellulitis, dermatology, information, mixed methods, patient experience, primary health care

## Abstract

**Background:**

Cellulitis is a painful infection of the skin and underlying tissues, commonly affecting the lower leg. Approximately one-third of people experience recurrence. Patients’ ability to recover from cellulitis or prevent recurrence is likely to be influenced by their understanding of the condition.

**Aim:**

To explore patients’ perceptions of cellulitis, and their information needs.

**Design and setting:**

Mixed-methods study comprising semi-structured, face-to-face interviews and a cross-sectional survey, recruiting through primary and secondary care, and advertising.

**Method:**

Adults aged ≥18 years with a history of cellulitis were invited to take part in a survey, qualitative interview, or both.

**Results:**

In all, 30 interviews were conducted between August 2016 and July 2017. Qualitative data highlighted a low awareness of cellulitis before the first episode, uncertainty about when it had been diagnosed, concern/surprise at the severity of cellulitis, and a perceived insufficient information provision. People were surprised that they had never heard of cellulitis and that they had not received advice or leaflets giving self-care information. Some sought information from the internet and found this confusing.

A total of 240 surveys were completed (response rate 17%). These showed that, although many participants had received information on the treatment of cellulitis (60.0%, *n* = 144), they often reported receiving no information about causes (60.8%, *n* = 146) or prevention of recurrence (73.3%, *n* = 176).

**Conclusion:**

There is a need to provide information for people with cellulitis, particularly in regard to naming their condition, the management of acute episodes, and how to reduce the risk of recurrences.

## INTRODUCTION

Cellulitis is a painful and potentially serious infection of the skin and underlying tissues that commonly affects the lower leg. It can have a considerable impact on patients due to pain and the need for elevation of the affected limb.^1^ Cellulitis also has a substantial impact on the NHS, with 123 644 patients being admitted to hospitals in England for cellulitis between 2015 and 2016.^2^

Approximately one-third of people with cellulitis suffer recurrent episodes.^3^^,^^4^ The only treatment shown to reduce the risk of recurrence is long-term antibiotics.^5^^,^^6^ Other potential strategies to prevent recurrence include using emollients for prevention of dry, cracked skin, checking for and treating tinea pedis, and managing lymphoedema using compression stockings.^5^ If patients do not have information about how to prevent recurrence, or do not have an understanding around causation of cellulitis, then they are unlikely to modify risk factors in order to prevent recurrent episodes. One small qualitative study has been carried out among people treated for cellulitis in secondary care. Participants spoke about a lack of information and support, and the fact that they were often not told what they could do to prevent recurrence.^1^ This may be due to uncertainties regarding how best to prevent cellulitis.

The authors conducted a mixed-methods study to explore patients’ views and experiences of cellulitis, beliefs about possible causes of cellulitis, views about potential methods of preventing recurrent episodes, and their information needs. This paper focuses on patients’ views of cellulitis and the information they received, or wish they had received, from their GP and other health professionals. The authors’ findings on patients’ perceived causes of cellulitis, and views about prevention of recurrence, are reported elsewhere.

## METHOD

A cross-sectional survey and semi-structured, face-to-face interviews were conducted to produce generalisable as well as in-depth findings regarding patients’ views and experiences of cellulitis.^7^

### Participants and recruitment

The authors invited all eligible participants (adults aged ≥18 years diagnosed with cellulitis in the past 6 months, or with ≥two episodes of recurrent cellulitis within the past 3 years) in South West England through mail-outs from 25 GP practices across Hampshire, Wiltshire, and Dorset, opportunistic recruitment in two NHS hospital trusts in Southampton and North Hampshire, and through community advertising (advertisement in local newspaper). GP practices were invited to express an interest in the study in response to an advert circulated by the Wessex Clinical Research Network (CRN). The authors then identified and recruited GP practices in rural and urban locations, and from areas of differing levels of social deprivation, according to the deprivation scores provided in the National General Practice profiles published by Public Health England,^8^ in order to identify participants with a range of perspectives. Participants could choose to take part in an interview or complete the survey, or both.

**How this fits in**

Cellulitis is a common infection of the skin that is often recurrent and necessitates substantial antibiotic use, with a negative impact on quality of life. Patients’ ability to recover from acute cellulitis, or potentially prevent recurrence, is hampered by their limited knowledge about the condition. Many patients reported a lack of information at point-of-care, and some had received conflicting advice from different health professionals. There is a need for clear advice for people with cellulitis, particularly informing them about the name of their condition, how to manage an acute episode (prompt help-seeking, rest, and elevation), and what they can do to reduce the risk of recurrence.

### Data collection

For the qualitative interviews, the authors purposively sampled participants for age, sex, recruitment source, and number of episodes of cellulitis. They sought written informed consent before carrying out interviews, which were conducted in participants’ homes (*n* = 26) or an alternative location of their choice, for example, a local coffee shop (*n* = 4). The authors used a semi-structured interview guide and, with participants’ permission, interviews were audio-recorded and transcribed verbatim. The interviews lasted from 35–90 minutes. The interview guide (Box 1) and questionnaire (available from the authors upon request) were developed by the research team by drawing on existing literature and input from patient collaborators. Participants who took part in an interview received a £10 voucher to reimburse them for their time. Eligible participants opting to take part in the survey could choose to complete a paper or identical online survey; 94.6% of participants (*n* = 227) completed the paper survey.

Box 1. **Interview topic guide**

**Table:** 

**About cellulitis**
1. Can you tell me a bit first of all about your experience of cellulitis? How many times have you had cellulitis? Where did it affect you (for example, the leg)? How much of an impact do you think having cellulitis had on you? Where diagnosed? Where treated?
2. What thoughts have you had about why you had cellulitis? Or why you might have been more at risk of having cellulitis?
3. What thoughts have you had about the possibility of cellulitis coming back again?
4. What do you think about your chance of having cellulitis compared with other people?
**About prevention**
1. Do you do anything to try to prevent cellulitis from coming back again? If so, what? Any barriers to this? How difficult to keep going with it? How did you find out about this? Have you ever tried anything in the past, or thought about trying anything to prevent getting cellulitis?
2. If we knew that it worked, how would you feel about doing something regularly to prevent cellulitis from coming back? Like putting cream on your feet every day? Or washing or drying your feet every day? Compression stockings Can you think of any potential problems with this? Do you have many other tasks related to your health that you have to do every day? If so, how do you find it managing them all? How would this fit in?

### Analysis

The authors conducted an inductive thematic analysis to explore the interview data.^9^ One author read the transcripts to familiarise herself with the data, and conducted line-by-line coding. Codes were derived inductively from the data, grouped together to produce an initial coding frame, and reviewed to identify similarities and differences. A detailed coding manual was created to ensure transparent and systematic coding of the data, and a negative case analysis carried out. Codes and theme/subtheme definitions were discussed with, and iteratively developed by, members of the research team to offer diverse inferences and interpretation of the data. The authors finished data collection once data saturation of the main themes was reached. Using NVivo (version 11) enabled a detailed audit trail to be maintained. The authors used SPSS (version 24) to provide descriptive statistical analysis of the survey data.

## RESULTS

One author conducted 30 semi-structured, face-to-face interviews between August 2016 and July 2017. Study sites distributed 1418 surveys, of which 236 were either completed online or paper copies returned to the research team in the freepost envelope provided (response rate 17%). A further four surveys were completed by participants recruited via community advertising. Participants’ characteristics are presented in Table 1.

**Table 1.** Participant characteristics (interviewees and survey responders)

	**Interviewees, N = 30 n (%)**	**Survey responders, N = 240 n (%)**
**Sex**		
Female	16 (53)	128 (53)
Male	14 (47)	103 (43)
Missing	0 (0)	9 (4)

**Age**		
18–25 years	0 (0)	2 (1)
26–45 years	4 (13)	14 (6)
46–65 years	9 (30)	80 (33)
66–75 years	11 (37)	71 (30)
76–85 years	6 (20)	53 (22)
>85 years	0 (0)	14 (6)
Missing	0 (0)	6 (3)

**Recruitment source**		
Primary care	16 (53)	216 (90)
Secondary care	8 (27)	20 (8)
Community advertising	6 (20)	4 (2)

**Number of cellulitis episodes**		
First episode	10 (33)	109 (45)
Recurrent episodes	20 (67)	130 (54)
Missing	0 (0)	1 (1)

**Location of cellulitis**		
Lower leg	25 (83)	170 (71)
Upper leg	1 (3)	6 (3)
Arm/hand	4 (13)	6 (3)
Face	0 (0)	9 (4)
Multiple locations	0 (0)	34 (14)
Elsewhere on the body	0 (0)	14 (6)
Missing	0 (0)	1 (0)

**Long-standing illness^a^**		
Yes	23 (77)	166 (69)
No	7 (23)	66 (28)
Missing	0 (0)	8 (3)

a Anything that has troubled participant over a period of time, or that is likely to affect participant over a period of time.

### Key themes

Thematic analysis of the interview data highlighted a general sense of uncertainty among the participants in terms of awareness and understanding of cellulitis, and their experiences of primary (first episode) cellulitis. Analysed data relating to uncertainty about cellulitis were coded into four key themes:

- low awareness of cellulitis before first episode;
- uncertainty about the time of diagnosis;
- concern/surprise at the severity of cellulitis; and
- perceived insufficient information provision.

The authors explore these themes in detail below, and present selected quotes to illustrate each theme.

#### Low awareness of cellulitis before first episode

Despite being a relatively common skin infection, interview data showed low awareness of cellulitis among participants before diagnosis. In discussing their experiences of being diagnosed with cellulitis for the first time, participants commonly talked about it as something previously unheard of. Many were surprised that they had never heard of cellulitis before diagnosis and, as such, felt that it was generally not a particularly common or well-known condition:

‘Is it a new thing, cellulitis? I’ve never heard of it before. Because it’s a serious infection; it can come up anywhere on your body.’

(Participant [P] 12, male [M], 83 years, first episode)

#### Uncertainty about the time of diagnosis

For some participants, this sense of uncertainty and unfamiliarity about cellulitis seemed to be further compounded by their experiences around the time of diagnosis. Though some participants expressed relatively straightforward experiences and received a clear diagnosis, many appeared to have more negative reports, including delayed diagnosis, no definitive diagnosis, and in some cases misdiagnosis. A commonly expressed view was that the health professionals seemed to be unsure about diagnosing cellulitis, and that diagnosis had often occurred following a second opinion:

‘I can remember going to see a doctor there in the practice in the local village. I think, I’m not sure if it was this guy I’m thinking of, it might have been the previous GP, who said it may have been cellulitis, he wasn’t sure. But I think the second GP I saw had a background in, I forget the technical name, said: “I think we ought to get your bloods checked.” And so he checked them and, I remember, he confirmed that it was cellulitis.’

(P23, M, 48 years, recurrent cellulitis)

Participants’ uncertainty around cellulitis also seemed to be fuelled by the lack of a definitive diagnosis. Some participants reported that, though they had been told they had a skin infection (and all participants had been invited on the basis of having received a diagnosis of cellulitis), their health professional had never told them that it was called cellulitis, even though they would have appreciated knowing exactly what infection it was:

‘I’d never even heard of it, as I say, until it was mentioned about in the study information. I thought, OK, interesting, cellulitis. Prior to that, I didn’t even know what it was; I just thought it was an infection. It would have been useful just to have been given a name.’

(P16, female [F], 32 years, first episode)

#### Concern/surprise at the severity of cellulitis

Many participants expressed concern and surprise about the potential severity of cellulitis, particularly in terms of its sudden onset, illness progression, and the long duration of symptoms and treatment. This is perhaps unsurprising given that, for the majority of participants, cellulitis was an unknown/unheard of condition before diagnosis. A common concern reported by participants was the sudden onset — that is, going from feeling slightly unwell to very unwell within a very short time:

‘Because it was so quick and so sudden. I went from being fine to — 12 hours later — heading for the hospital, so it was so quick. Apart from being quite traumatic, I was off work for about 6 weeks, and I think mentally it got me a bit. I mean, I’ve never been that ill before, and it actually worried me a bit.’

(P4, M, 61 years, recurrent cellulitis)

*‘And, really, the speed of it. I got an emergency appointment on the Wednesday and it was just getting worse, and on the Friday, that was me in* [hospital]*. So, it was a couple of days and went from 0 to bloody hell in a few days.’*

(P15, M, 63 years, recurrent cellulitis)

Another common concern or cause for surprise was realising that cellulitis is a potentially serious condition and there is no quick fix. Participants expressed concerns about potentially severe consequences of illness progression and spread of infection, as well as length of time to recovery and the long duration of treatment. In particular, many participants spoke about having to take several courses of antibiotics and/or large dosages of antibiotics and experiencing symptoms for several weeks:

“As I say, that’s — it took — you could say it’s taken — from the total — 4 weeks at least for cellulitis, to be told that it was nearly gone or even maybe longer than that. I mean, even going past the holiday, 6–8 weeks and you just think, it’s taken all that time, I suffered for all that time.’

(P17, F, 60 years, recurrent cellulitis)

‘Doctor gave me a dose of 7 days antibiotics, a higher dosage. This was after the 5 days antibiotics. She said: “On these ones, you should see an improvement within 48 hours.” That didn’t happen, so I went straight up to A&E. They did all the blood tests and they come back and they said: “Yes, you’ve got a really severe case of cellulitis.” Stuck me on a drip of antibiotics and a drip of penicillin for 3 days. Every day, for 3 days, I had to go back at 3 o’clock in the afternoon, get hooked up on these drips for half an hour each time.’

(P1, M, 47 years, first episode)

#### Perceived insufficient information provision

The interviews showed patients felt they were not given information on causes for cellulitis, or treatment and prevention, which seemed to exacerbate participants’ uncertainty about cellulitis. Most participants reported they had received little or no information at point-of-care, particularly around the potential causes of cellulitis, the fact that people can experience repeat episodes of cellulitis, and potential strategies to prevent recurrence. For some participants, this perceived lack of information seemed to be linked to having not received an official diagnosis of cellulitis:

‘I didn’t even know you can get it again and again; you told me that. I thought you get it once and then it’s gone, you won’t get it again. I’ve only learned you can get a recurrence through you, which the doctor should tell you, really.’

(P11, M, 53 years, first episode)

‘I was wearing boots, and where they touched it, it was really, really tender, and that was when he said it was cellulitis. And he gave me antibiotics, but that’s all he said. He said “it’s a skin infection”, and — and that was it. I don’t know why I had it or … and then I got it back again, that was about, probably about a year ago.’

(P5, F, 67 years, recurrent cellulitis)

The lack of information provided by health professionals led some participants to seek out their own information about cellulitis from other sources, such as friends/colleagues and online resources, but many expressed doubts about the credibility of online information:

*‘I received absolutely no information. As I say, it was when the research nurse came along and said: “Oh, we’re doing the study.” I went: “Oh, cellulitis, I’ve never even heard of it.” And then it was really me Googling it and trying to find out more information. If you put a Google search in for it, you get a lot of beauty treatments for cellulite* [laughter] *and I was thinking, “Oh, is it linked to this?” But it must be something to do with your skin because of that. But trying to actually find good, sort of basic information on the net was really difficult as well.’*

(P19, F, 53 years, first episode)

Participants who did receive advice on managing symptoms and preventing recurrence expressed concerns about the conflicting nature of this advice — for instance, about limb elevation (rest versus exercise) and about prevention (foot hygiene). This is perhaps unsurprising, given the lack of evidence around cellulitis:

‘The one thing the doctor didn’t do, which I — was kind of a mixed message — he said, I must rest my legs up as much as possible, but didn’t say “so, I’m going to sign you off work”. So, as a consequence, I was teaching, which involves standing up all the time. So, I was probably exacerbating it and making it last a lot longer, because I didn’t — I wasn’t told that it was something that I should have time off for.’

(P2, M, 45 years, recurrent cellulitis)

‘And I kept getting conflicting advice as well. One doctor would say “keep your leg up”, the other doctor would say “go and do some exercise”.’

(P13, F, 32 years, recurrent cellulitis)

A desire for detailed and reliable information on cellulitis prevention provided by GPs or other health professionals was evident in the data. Many participants expressed a desire for more information in the form of a leaflet or website. Some participants reflected on the perceived lack of cellulitis information provided to patients, compared with other chronic and acute health conditions:

‘Well, I think — information — maybe not so much as how you probably got it, because there’s probably different — I mean, I’ve looked it up on the internet; you can get it through lots of different things, but maybe a bit more information, the care during, while I had it, and also the care for afterwards. Maybe you’re a person more prone to get it, you may get it again; these are the things you can do to prevent it, whatever, because if you don’t know what you’re doing and you do get it again, you’re wasting their time. My partner had pneumonia earlier last year and he got a leaflet about pneumonia, so why can’t they do one on cellulitis?’

(P17, F, 60 years, recurrent cellulitis)

### Survey data

The survey asked participants whether they had received any information about the causes, treatment, and prevention of cellulitis (Table 2). Only 60% (*n* = 144) of participants reported that they were given information on the treatment of cellulitis. Most were given no information about the causes of cellulitis (60.8%, *n* = 146), or about how to prevent recurrence (73.3%, *n* = 176). Of those who did receive any information about their cellulitis, nearly half felt the information was insufficient and did not meet their needs (Table 3).

**Table 2.** Information received on cellulitis causation, treatment, and prevention

	**Number of participants (N = 240)**	**Percentage**
**Information on causes**		
Yes	90	37.5
No	146	60.8
Missing	4	1.7

**Information on treatment**		
Yes	144	60.0
No	93	38.8
Missing	3	1.3

**Information on prevention**		
Yes	56	23.3
No	176	73.3
Missing	8	3.3

**Table 3.** Perceptions of information received about cellulitis causation

	**On causation**		**On treatment**		**On prevention**	
	**Participants, *n* = 90/290**	**%**	**Participants, *n* = 144/290**	**%**	**Participants, *n* = 56/290**	**%**
I received some information, but I would have liked more	37	41.1	69	47.9	23	41.1
I felt informed, but found my own information from other sources	26	28.9	23	15.9	16	28.6
I felt fully informed by the healthcare professionals	26	28.9	43	29.9	16	28.6
Missing	1	1.1	9	6.3	1	1.8

## DISCUSSION

### Summary

The diagnosis, treatment, and prevention of cellulitis appears to be poorly understood by patients. Participants reported low awareness of the condition before diagnosis, and uncertainty about the time of diagnosis. The quantitative data suggest that patients perceived they are generally not given sufficient information about cellulitis by health professionals, particularly in terms of causation and prevention. Participants in the qualitative study also highlighted insufficient information around cellulitis, contrasting this with information that they had received for other medical conditions. Participants particularly perceived a lack of information provision at time of diagnosis, and confusing and conflicting information online and offline.

### Strengths and limitations

This is the first study that the authors are aware of that thoroughly explores patients’ concerns regarding perceptions of cellulitis and their information needs. A strength of the authors’ approach is that by using both qualitative interviews and quantitative survey methods they are able to examine experiences in detail, while also providing estimates that generalise about the number of patients with unmet information needs. A limitation is the relatively low response rate to invitations to participate, meaning that these views may not be representative of all people with a history of cellulitis.

### Comparison with existing literature

The qualitative study by Carter *et al* 1 mainly focused on patients’ perspectives of service delivery for cellulitis, but some data suggest mixed experiences in terms of information provision and fear of the potential for recurrence. In common with the present study, they also found that patients were sometimes unaware of their diagnosis, and suggested the need for information leaflets. The current study’s finding that patients would prefer to be signposted towards reliable information echoes other research suggesting that patients are likely to seek information online in order to address offline knowledge deficits.^10^ Others have found that the breadth of information available online is potentially bewildering, particularly as all sources of information might be seen as having equivalent status, regardless of their trustworthiness.^11^ Cellulitis is unusual in that it is both an acute event and a potentially chronic illness for those who experience recurrent episodes, though many patients appear unaware of this. Clear and consistent information should be made available to patients (online and offline) about how to manage an acute episode, with suggestions of possible strategies for the prevention of recurrence.

### Implications for research and practice

Some patients reported that they had received conflicting advice, both about treatment and prevention. This reflects evidence gaps and the need for trials of treatment and prevention strategies in order to inform the development of clear, consistent, reliable information and advice for health professionals and patients. The need to determine the best non-antibiotic intervention for the prevention of cellulitis was highlighted in a recent James Lind Alliance Cellulitis Priority Setting Partnership.^12^ The authors of the current study examined currently available online patient resources about causes, treatment, and prevention of cellulitis (Box 2), and found inconsistencies between sources. For example, the need to seek medical advice urgently differs between sources, which may lead to confusion and poor outcomes, and more work should be done to address these inconsistencies. Given the uncertainty around diagnosis highlighted by cellulitis patients in this study, future research exploring the views and experiences of healthcare professionals in relation to diagnosing cellulitis and providing information on it may be beneficial.

Box 2. **Examples of sources of information for people with cellulitis**

**Table:** 

**Website**	**URL**
**NHS Choices**	http://www.nhs.uk/conditions/Cellulitis/Pages/Introduction.aspx
**Patient.co.uk**	https://patient.info/health/cellulitis-and-erysipelas-leaflet
**British Association of Dermatologists**	http://www.bad.org.uk/for-the-public/patient-information-leaflets
**Lymphoedema Support Network**	http://www.lymphoedema.org/index.php/information-for-health-care-professionals/rcgp-module

The authors’ findings suggest the need for clear communication of basic information for people with cellulitis, particularly in regard to being informed of the name of their condition, how to manage an acute episode (prompt help seeking, rest, and elevation of the affected limb), and what patients can do to reduce the risk of recurrence. Consistent information from health professionals, or signposting towards reliable, credible information, would be welcomed.
